# GARCOM: A user-friendly R package for genetic mutation counts

**DOI:** 10.12688/f1000research.53858.1

**Published:** 2021-07-01

**Authors:** Sanjeev Sariya, Giuseppe Tosto

**Affiliations:** 1 Taub Institute for Research on Alzheimer’s Disease and the Aging Brain, Columbia University Medical Center, New York, NY, 10032, USA; 2 The Gertrude H. Sergievsky Center College of Physicians and Surgeons, Columbia University Medical Center, New York, NY, 10032, USA; 3 Department of Neurology College of Physicians and Surgeons, Columbia University Medical Center, New York, NY, 10033, USA

**Keywords:** mutation, plink, allele, genetics, VCF

## Abstract

Next-generation sequencing (NGS) has enabled analysis of rare and uncommon variants in large study cohorts. A common strategy to overcome these low frequencies and/or small effect sizes relies on collapsing strategies, i.e. to bin variants within genes/regions. Several tools are now available for advanced statistical analyses however, tools to perform basic tasks such as obtaining allelic counts within defined genetics boundaries are unavailable or require complex coding. GARCOM library, an open-source freely available package in R language, returns a matrix with allelic counts within defined genetic boundaries. GARCOM accepts input data in PLINK or VCF formats, with additional options to subset data for refined analyses.

## Introduction

Genome-wide association studies (GWAS) have led to the identification of several genomic common variants associated with complex diseases, ^1^ yet missing heritability remains extensive. Furthermore, most of the disease-causing variants are rare in nature ^2^ where common variants serve as a proxy. Rapid decline in sequencing costs have enabled in-depth analysis of rare variants (RVs; minor allele frequency < 1%) through Whole-Genome sequencing (WGS) and Whole-Exome Sequencing (WES). Furthermore, large-scale reference panels have allowed for imputation of RVs. ^3^^-^^5^ Power to identify statistically significant RVs decreases as the minor allele frequency decreases: therefore, an ideal method to overcome this limitation is to group RV at the gene/region level, usually via a collapsing test.

Despite the availability of sophisticated tools for annotation, quality-control and association analyses, tools to perform basic tasks, for instance, obtaining allelic count within defined genetic boundaries (genes and/or regions) are lacking, to our knowledge. R libraries such as BEDMatrix and bigsnpr ^6^ provide allelic counts for each SNP per individual but algorithms to extract information within genetic boundaries in a collapsed fashion are unavailable.

Here we introduce a user-friendly R package, GARCOM (“ **G**enetic **A**nd **R**egional **C**ount **o**f **M**utations”) that provides allelic counts per individual within user-provided genetics/regional boundaries.

## Methods

GARCOM is written and developed in open-source R ^7^ statistical and programming language. GARCOM imports *data.table*, ^8^ *vcfR*, ^9^ *bigstatr*, *bigsnpr* and *stats* libraries for internal data transformation and processing. A stable version is released and publicly available on the CRAN repository.

**install.packages(“garcom”)**

### Operation

GARCOM was developed on R (≥4.0) (RRID:SCR_017299) with other dependencies and minimum versions as: data.table (≥1.12.8), vcfR (≥1.12.0), bigsnpr (≥1.4.11). Full documentation of dependencies and installation is available at GARCOM github repository. There is no minimum memory (RAM) requirement as far as we know, but that may vary according to the nature and size of input genetics data. GARCOM was developed on Unix platform but can also be used on other platforms (e.g. Windows, Ubuntu).

### Implementation

GARCOM operates through two main functions: “ *gene_pos_counts*” accepts PLINK ^10^ (RRID:SCR_001757) input data, whereas “ *vcf_counts_SNP_genecoords*” accepts VCF ^11^ input format. After reading in the data, these functions perform operations to count variants within genes/genomic regions for each individuals.

**output <- gene_pos_counts(recoded_genetic_data, gene_boundaries, snp_locations)**

**output <- vcf_counts_SNP_genecoords(recoded_genetic_data, gene_boundaries, snp_locations)**

where, “ *output*” is the object generated by our library after a successful run of function; “ *recoded_genetic_data*” is the main input file in PLINK or VCF formats; “ *gene_boundaries*”, and “ *snp_locations*” are additional input files for gene and SNP information, respectively.

**Figure 1.**
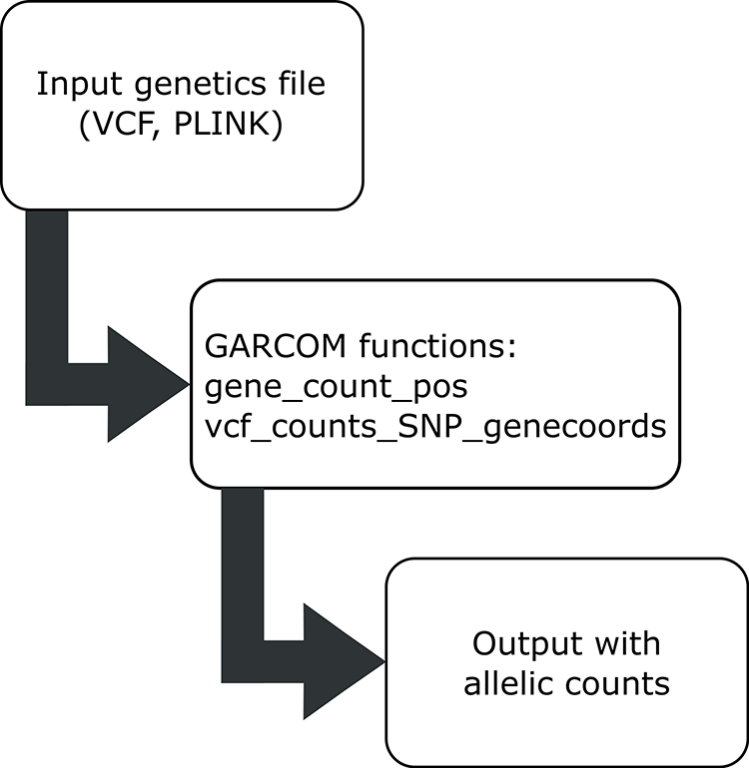
Workflow for standard GARCOM functions. GARCOM provides functions to read output from PLINK, VCF format. The final output is a matrix with rows as gene names and columns containing individuals’ IDs.

Typical workflow is shown in Figure 1. In brief, *the “gene_pos_counts*” function will process genetic input data (“ *recoded_genetic_data”*) generated from the PLINK software through the --recode A option. Data are read in standard matrix format using the *data.table* R library. For VCF files, the “ *vcf_counts_SNP_genecoords”* function reads the VCF input file employing the *extract.gt* function from the *vcfR* library. The genotype values are read within the “GT” field.

In addition to the --recode A genetic input, GARCOM needs genetic boundaries information and SNP information as shown in Table 2 and Table 3, respectively.

Output produced by GARCOM is a matrix, with *M* rows and *N* columns, where *M* represents the genes/genomic regions with at least one allele count and *N* represents the individuals. Genes with zero allelic counts across all individuals are excluded from the final output. Missing values are counted as zero in final output. When no allelic counts are present in the user-defined genes, NULL value is returned. GARCOM allows missing values (NA) in input data.

In addition to the function described, GARCOM provides several options for user flexibility. For instance, GARCOM can be ran restricting analyses to 1) a list of genes, or 2) filter SNPs and extract individuals of interest. For instance, users can provide list of individuals using the “ *keep_indiv”* parameter; similarly, genes can be filtered in by using the “ *filter_gene*” parameter.

**output <- gene_pos_counts(recoded_genetic_data, gene_boundaries, snp_locations,** *keep_indiv=mylist.txt*)

**output <- gene_pos_counts(recoded_genetic_data, gene_boundaries, snp_locations,** *filter_gene=mysetofgenes.txt*)

### Use cases

The input PLINK file has a matrix structure of *N* rows with *M* columns, where *N* rows represent individuals (one for each ID). The first six columns are family ID, individual ID, paternal ID, maternal ID, sex, and phenotype (standard output from PLINK ( Table 1)). Following columns consist of the variants included in the analyses.

**Table 1.** Sample rows and columns for input genetics data recoded from PLINK software (--recode A).

FID	IID	PAT	MAT	SEX	PHENOTYPE	SNP1_A	SNP2_T	SNP3_G	SNP4_C	SNP5_C
FID1	IID_sample1	0	0	1	NA	1	1	0	NA	NA
FID2	IID_sample2	0	0	1	NA	0	1	0	NA	0
FID3	IID_sample3	0	0	1	1	0	0	1	0	0
FID4	IID_sample4	0	0	1	1	0	0	1	0	0
FID5	IID_sample5	0	0	1	1	0	0	1	0	0

**Table 2.** Sample data for genetics boundaries. Data must contain GENE, START and END column names.

GENE	START	END
GENE1	100	180
GENE2	200	400

**Table 3.** Sample data for SNP information where SNP and BP column names are must in input data, where SNP is single nucleotide polymorphism or variant and BP is base pair location.

SNP	BP
SNP1	100
SNP2	101
SNP3	201

**Table 4.** Sample output, where GENE column consists of gene names with corresponding individuals. Individual_ID1, Individual_ID2 and Individual_ID3 are sample individual IDs, where values represent allelic counts within gene for individual.

GENE	Individual_ID1	Individual_ID2	Individual_ID3
GENE1	10	2	1
GENE2	2	1	0

The input VCF file follows the standard VCF formats (please refer to the vcfR library documentation).

Toy data (gene and SNP coordinates) are shared within the package as “ *genecoord*” and “ *snppos*”, respectively.

### Simulation

**Figure 2.**
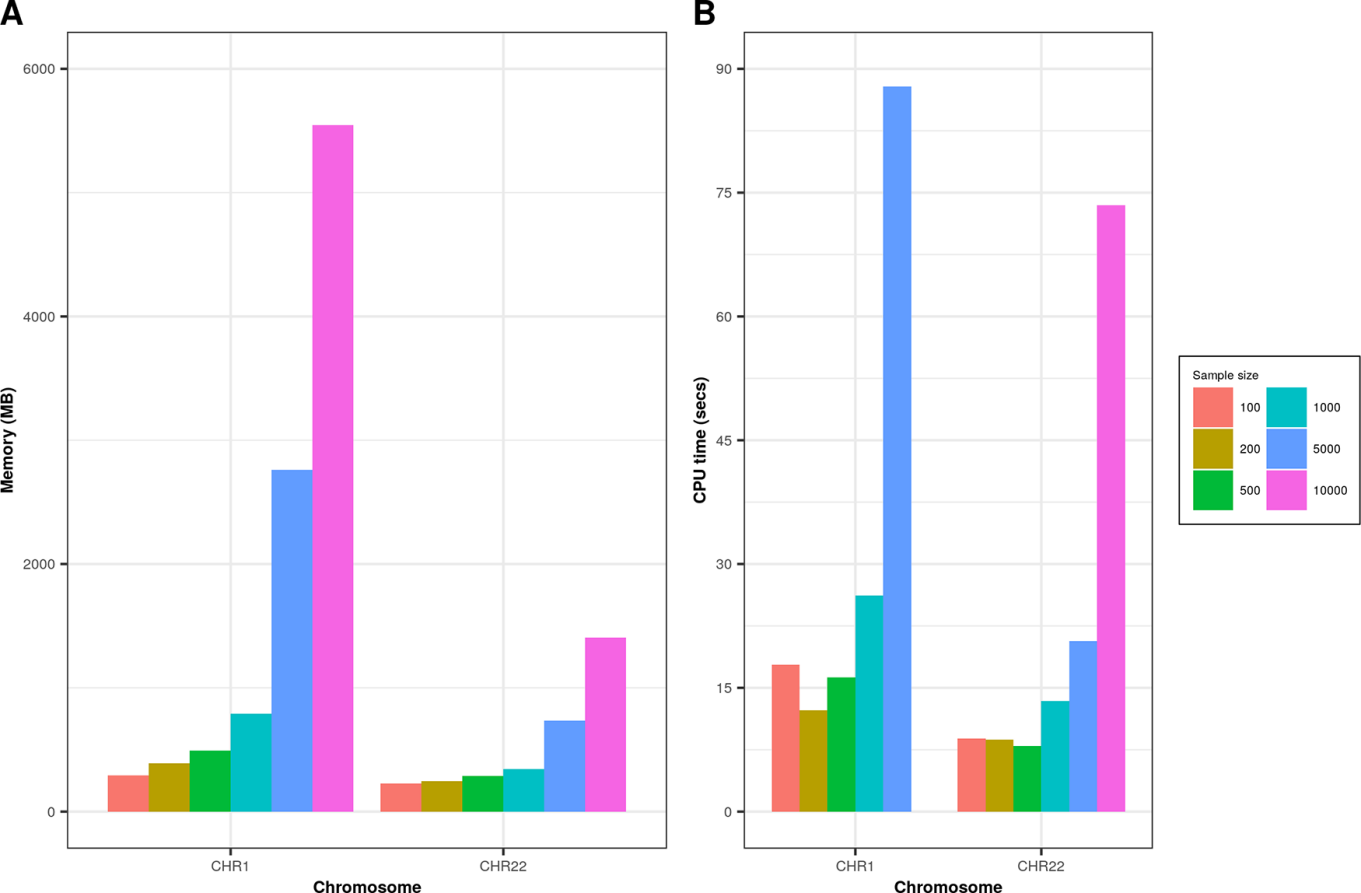
Comparison of memory (in MB) and CPU time (in seconds) for CHR1 and CHR22 on different sample sizes. Graph A represents memory consumption; graph B shows processing time in seconds for various sample sizes on CHR1 and CHR22.

We performed simulation on real data for CHR1 (# of variants = 23,456) and CHR22 (# of variants = 4,814) on randomly sampled individuals (N = 100, 200, 500, 1000, 5000, 10,000) extracted from whole-exome sequencing dataset as described in the study by Tosto et al. ^12^ Genetics data were recoded using PLINK --recode A flag. On both chromosomes we found increased memory consumption and time ( Figure 2) as we increased the number of individuals processed. Memory consumption for CHR22 was significantly lower than CHR1 due to a smaller number of variants and genomic boundaries. Simulations were performed with 16GB memory (RAM) requested on computing cluster node.

All simulations were conducted on R (v4.0), data.table (v1.13.6) with default 16 threads, GARCOM (v1.40), bigsnpr (v1.6.1).

## Discussion

GARCOM is easy to use where basic knowledge of R programming language is helpful but not desired. GARCOM is designed by harnessing existing libraries, such as *data.table,* that allow for efficient handling of large data. GARCOM data processing is independent of the reference genome build. GARCOM can be used on several platforms (e.g., Unix, Windows). GARCOM comes with certain limitations: genomic boundaries and variants' location need to be specified, as mentioned in the package documentation. In case of large-sized studies, for example UK biobank (N ≥ 200K), processing data per chromosome is highly recommended due to memory limitations. Lastly, GARCOM depends on public and freely available R packages.

### Future

VCF format can accommodate locus annotation performed by software such as ANNOVAR. ^13^ To this end, GARCOM plans to accommodate annotation filters in addition to the existing ones. One challenge associated with annotated VCF is the resulting large file size; we will try to add this functionality, keeping RAM limitations and processing time in mind. We plan to add features to handle bgen format which stores large amount of genetics data, with appropriate R library ( http://www.well.ox.ac.uk/~gav/resources/rbgen_v1.1.5.tgz).

## Data and software availability

Sample data associated with the package where applicable are provided within the library with proper documentation. No additional source data are required. We distribute the package under the MIT license. GARCOM can be downloaded from CRAN and GitHub from https://cran.r-project.org/web/packages/GARCOM/index.html and https://github.com/sariya/GARCOM respectively.

**Reporting guidelines**: Bugs and suggestions are welcome at the GitHub repository.

**Author Contribution**: SS, GT

**Ethical Statement**: Informed consent was obtained from all participants. For the whole-exome sequencing, the study protocol was approved by the Institutional Review Board (IRB) of Columbia university Medical Center (CUMC) (Approval number: AAAP0477). The study was conducted according to the principles expressed in the Declaration of Helsinki.
