# Supplementary material for: GARCOM: A user-friendly R package for genetic mutation counts
Source: F1000Res. 2024 May 17;10:524. Originally published 2021 Jul 1. [Version 2] doi: 10.12688/f1000research.53858.2 (PMC11904396; doi:10.12688/f1000research.53858.2)
Supplement: Peer review history

# Reviewer report: Reviewer response for version 2

**DOI:** 10.5256/f1000research.166261.r365266

**Published:** 2025-03-12

**Authors:** Tao Wang

**Affiliations:** 1 School of Computer Science, Northwestern Polytechnical University, Xi’an, China

In this work, the authors developed an R package which could retrive the allelic counts within a genomic region. I have the following suggestions:

 As the genomic files are usually very large, it is necessary to take care of memory usage and running time when dealing with large files.

 Besides, the reference of related works should be added in the backgound, including its potential to benefit the genetic data QC and downstream analyses like QTL, SNV analysis.

Are the conclusions about the tool and its performance adequately supported by the findings presented in the article?

Yes

Is the rationale for developing the new software tool clearly explained?

Yes

Is the description of the software tool technically sound?

Yes

Are sufficient details of the code, methods and analysis (if applicable) provided to allow replication of the software development and its use by others?

Yes

Is sufficient information provided to allow interpretation of the expected output datasets and any results generated using the tool?

Yes

Reviewer Expertise:

genetics, machine learning

I confirm that I have read this submission and believe that I have an appropriate level of expertise to confirm that it is of an acceptable scientific standard.

## References

[1] : A pipeline for RNA-seq based eQTL analysis with automated quality control procedures. *BMC Bioinformatics* .2021;22(Suppl 9) : 10.1186/s12859-021-04307-0 403 10.1186/s12859-021-04307-0 34433407 PMC8386049

[2] : Enhancing discoveries of molecular QTL studies with small sample size using summary statistic imputation. *Brief Bioinform* .2022;23(1) : 10.1093/bib/bbab370 10.1093/bib/bbab370 34545927

[3] : stSNV: a comprehensive resource of SNVs in spatial transcriptome. *Nucleic Acids Res* .2025;53(D1) : 10.1093/nar/gkae945 D1224-D1234 10.1093/nar/gkae945 39470702 PMC11701523

# Reviewer report: Reviewer response for version 2

**DOI:** 10.5256/f1000research.166261.r279700

**Published:** 2024-06-14

**Authors:** Ettore Mosca

**Affiliations:** 1 National Research Council, Institute of Biomedical Technologies, Segrate (Milan), Italy

The authors amended the manuscript and answered to my comments. I have no further comments.

Are the conclusions about the tool and its performance adequately supported by the findings presented in the article?

Partly

Is the rationale for developing the new software tool clearly explained?

Yes

Is the description of the software tool technically sound?

Partly

Are sufficient details of the code, methods and analysis (if applicable) provided to allow replication of the software development and its use by others?

Partly

Is sufficient information provided to allow interpretation of the expected output datasets and any results generated using the tool?

Partly

Reviewer Expertise:

Bioinformatics, NGS data analysis, network analysis, pathway analysis, metabolomics data analysis.

I confirm that I have read this submission and believe that I have an appropriate level of expertise to confirm that it is of an acceptable scientific standard.

# Reviewer report: Reviewer response for version 1

**DOI:** 10.5256/f1000research.57282.r98677

**Published:** 2021-11-29

**Authors:** Stephen M. Pederson

**Affiliations:** 1 Dame Roma Mitchell Cancer Research Laboratories, Adelaide Medical School, University of Adelaide, Adelaide, SA, Australia

The authors have provided four primary functions for summarising SNP counts using regions or genes as mapping architecture. The *data.table* package is known for its speed and this may lend a significant performance advantage to these functions, particularly when dealing with large numbers of samples or SNPs.

 However, as it stands, this submission appears to fall short in a few key areas. Firstly, there is a typo in the installation instructions: **install.packages(“garcom”)** will cause an error. The correct instructions should be **install.packages(“GARCOM”)**.

 The authors seem unaware of significant pre-existing infrastructure for range-based and SNP-based operations. The Bioconductor community has a wide-ranging suite of packages for robust mapping between genes, ranges, and SNPs, and no performance comparison, nor acknowledgment of these packages has been made. I suspect that using *data.table* may lend significant performance advantages, but the robustness of mapping between ranges provided by *GenomicRanges* is advantageous when performing rigorous research. For example, the lack of insistence on a CHR column for the *dt_gene *argument of *gene_pos_counts()* raises the distinct possibility of erroneous mapping between SNPs and genes/regions, and this should be addressed for robustness.

 The authors also seem unaware of other packages which deal very efficiently with PLINK and VCF data, such as *seqArray *and *snpRelate. *In particular, these packages utilise the *gdsfmt* infrastructure, which is extremely efficient at working with large numbers of SNPs and samples. How does GARCOM compare by using the *data.table* backend? The provision of a new, lesser performing package will serve minimal purpose and this becomes an important question.

 Whilst stating that the argument *dt_gene* follows PLINK format, an example of importing PLINK data would be most helpful.

 A simple example vcf should also be included in the package so that examples for the two vcf-based functions can be run by any potential users. An example with 10 SNPs and 10 individuals, as per the examples for *gene_annot_counts* and *gene_pos_counts,* would fall within size limits for an R package. The fact that this has not been done also highlights the fact that the two vcf-based functions have not been included in the *test_that* suite of tests, rendering them open to future or even current bugs.

 It is clear that the authors have found this package to be very useful for their work and their enthusiasm for making it available is commendable. However, I fear that the documentation is not adequate enough to invite new users. A *pkgdown* vignette appears to have been generated on the github repository but I could not find this vignette visible available anywhere and am unaware of its contents beyond skimming the visible HTML code. Please enable viewing of this via github.io pages which will take a matter of seconds. Making this visible (see https://sa-lee.github.io/plyranges for an excellent example), should enable new users to better comprehend the motivation and use of these functions. This would also enable the authors to include test coverage and other informative metrics.

 I also note that the default behaviour of *gene_pos_counts()* is to drop genes with zero counts across all individuals, instead of returning all input genes. This is not stated anywhere in the documentation and should be, or alternatively, an argument allowing this behaviour to be modified should be added. Zero counts is not a non-result. It is easy to imagine scenarios where this is indeed important information.

 The example data provided is also a little confusing. SNP1 in the object *snpgene* is mapped to both GENE1 and GENE4, and this relationship is not maintained in the *snppos* object. Is this meant to be SNP10 mapping to GENE4, or are the authors trying to highlight that multiple SNP to gene mappings are handled well by this package? If the latter, this may be a strong feature of the package and more detail should be provided for the handling of multi-mapping in the documentation. If, however, it is an oversight, this should be corrected so as not to confuse any future users.

 As to the F1000 submission, I would strongly encourage the inclusion of executed code where possible, with results from actual data objects so readers can easily see what each function is doing. As it stands, users will have to install the package locally and run the examples to really understand what the package is offering. Given the lack of example vcf or parsing of PLINK objects, this may prove an insurmountable hurdle for some. If clear examples are provided, I suspect this will also increase the uptake of the package by other users, which is an important consideration that I'm sure the authors wish to see.

Are the conclusions about the tool and its performance adequately supported by the findings presented in the article?

No

Is the rationale for developing the new software tool clearly explained?

Partly

Is the description of the software tool technically sound?

Partly

Are sufficient details of the code, methods and analysis (if applicable) provided to allow replication of the software development and its use by others?

No

Is sufficient information provided to allow interpretation of the expected output datasets and any results generated using the tool?

No

Reviewer Expertise:

Bioinformatician, R Package Developer

I confirm that I have read this submission and believe that I have an appropriate level of expertise to confirm that it is of an acceptable scientific standard, however I have significant reservations, as outlined above.

# Reviewer report: Reviewer response for version 1

**DOI:** 10.5256/f1000research.57282.r95650

**Published:** 2021-10-18

**Authors:** Ettore Mosca

**Affiliations:** 1 National Research Council, Institute of Biomedical Technologies, Segrate (Milan), Italy

The authors present GARCOM, a tool for quantifying allelic counts within defined genetic boundaries. Overall, the article is well-written; the software is available in CRAN and GitHub, and it is well-documented. At the same time, the article is quite short.

 I recommend adding some details to clarify how the proposed counting process works (e.g. it does not appear to keep track of the different haplotypes in different individuals) and its usefulness for genetic studies. This would clarify in which scenario this tool can be used and which kind of research questions it helps to address.

 In the proposed examples, genetic coordinates are given without chromosomes. This suggests that the tool handles one chromosome at a time. Even if this is the case, it might be a dangerous approach, because it could increase the probability of mixing genetic coordinates from different chromosomes while the user prepares the input for GARCOM.

Are the conclusions about the tool and its performance adequately supported by the findings presented in the article?

Partly

Is the rationale for developing the new software tool clearly explained?

Yes

Is the description of the software tool technically sound?

Partly

Are sufficient details of the code, methods and analysis (if applicable) provided to allow replication of the software development and its use by others?

Partly

Is sufficient information provided to allow interpretation of the expected output datasets and any results generated using the tool?

Partly

Reviewer Expertise:

Bioinformatics, NGS data analysis, network analysis, pathway analysis, metabolomics data analysis.

I confirm that I have read this submission and believe that I have an appropriate level of expertise to confirm that it is of an acceptable scientific standard, however I have significant reservations, as outlined above.

# Response

**Published:** 2021-11-15

**Authors:** Sanjeev Sariya

**Affiliations:** Columbia University Medical Center, USA

**We would like to thank the reviewer for providing feedback and reviewing our manuscript.**

 1) The authors present GARCOM, a tool for quantifying allelic counts within defined genetic

 boundaries. Overall, the article is well-written; the software is available in CRAN and GitHub, and it is well-documented. At the same time, the article is quite short.

**Reply:** The main goal of the manuscript is to provide a succinct description of R library, implementation, workflow along with a comparison with available methods. Contrary to most R packages, GARCOM performs one specific task (counting mutation/alleles per gene per sample), hence the brief manuscript.

 2) I recommend adding some details to clarify how the proposed counting process works (e.g. it does not appear to keep track of the different haplotypes in different individuals) and its usefulness for genetic studies. This would clarify in which scenario this tool can be used and which kind of research questions it helps to address.

**Reply:** The goal of GARCOM package is to perform allele counts within user-defined genetic region, GARCOM does not account for the haplotypes in different individuals. Allele counting is performed by simple arithmetic operation (sum)on user-provided single nucleotide polymorphisms (SNP) within the user-defined genetic boundaries. The main scope is mentioned in the manuscript, i.e. facilitate common yet laborious tasks for genetic studies, such as shaping files for collapsing analyses.

 3) In the proposed examples, genetic coordinates are given without chromosomes. This suggests that the tool handles one chromosome at a time. Even if this is the case, it might be a dangerous approach, because it could increase the probability of mixing genetic coordinates from different chromosomes while the user prepares the input for GARCOM.

**Reply:** Due to the increasing volume of genomics data, it has become challenging to process data genome-wide. GARCOM is therefore developed to process one chromosome at a time. Second, GARCOM collapses SNPs based on base-pair (BP) location, hence, processing data with multiple chromosomes would add additional burden (time and memory) and can be hazardous because of different SNPs with identical BP across the genome.
